# The effect of *IL10* gene polymorphism on obesity parameters in highly physically active young men

**DOI:** 10.5114/biolsport.2023.118336

**Published:** 2022-09-05

**Authors:** Ewelina Maculewicz, Andrzej Mastalerz, Bożena Antkowiak, Oktawiusz Antkowiak, Aleksandra Garbacz, Ewa Szarska, Paweł Rusin, Anna Cywińska, Agnieszka Białek, Paweł Cięszczyk

**Affiliations:** 1 Faculty of Physical Education, Jozef Pilsudski University of Physical Education in Warsaw, 00-968 Warsaw, Poland; 2 Military Institute of Hygiene and Epidemiology, 01-163 Warsaw, Poland; 3 Warsaw University of Life Sciences – SGGW, 02-787 Warsaw, Poland; 4 Institute of Biological Sciences, Cardinal Stefan Wyszynski University in Warsaw, 01-938 Warsaw, Poland; 5 Faculty of Biological and Veterinary Sciences, Nicolaus Copernicus University in Torun, 87-100 Torun, Poland; 6 Institute of Genetics and Animal Biotechnology of the Polish Academy of Sciences, 05-552 Magdalenka, Poland; 7 Faculty of Physical Education, Gdansk University of Physical Education and Sport, 80-336 Gdansk, Poland

**Keywords:** Interleukin, IL10, Genetic polymorphisms, Haplotype, BMI, Fat percentage

## Abstract

The main aim of this study was to investigate the association between 5 polymorphisms of the interleukin 10 (*IL10*) gene and body composition parameters in physically active young men. A cohort of 131 young men was enrolled and the following *IL10* single-nucleotide polymorphisms (SNPs) were analysed: rs1518111, rs1878672, rs3024496, rs3024498 and rs3024505. The subjects were divided into groups depending on obesity parameters: body mass index (BMI) and percentage of body fat tissue (fat %). Statistical analysis was conducted for alleles, genotypes and haplotypes, and an association between SNPs and body composition parameters was analysed using four genetic models: dominant, recessive, codominant and overdominant mode of inheritance (MOI). The only statistically significant result in polymorphisms was found for rs3024505 in the over-dominant model with BMI (p = 0.04) and with fat % (p = 0.02). The haplo.score function showed an association between BMI and CCGTA (respectively) haplotype in the additive model (score = -2.00, p = 0.04) and in the dominant model (score = -2.30, p = 0.02). The obtained results indicate a statistically significant contribution of selected *IL10* polymorphisms in the regulation of body weight in physically active individuals.

## INTRODUCTION

The World Health Organization (WHO) currently describes overweight and obesity as abnormal or excessive fat accumulation that may impair health. It is identified by the body mass index (BMI), obtained by dividing a person’s weight in kilograms by the square of height in meters (kg/m^2^). An overweight adult individual is one whose BMI is equal to or greater than 25, whereas an obese individual is one whose BMI is equal to or exceeds 30. Prevalence of obesity nearly tripled between 1975 and 2016. In 2016, according to WHO data, 39% of adults aged 18 years and over and 13% of the world’s adult population were overweight or obese, respectively [1]. The main cause of obesity and overweight is an energy imbalance between calories consumed and calories expended. Obesity is associated with a chronic low-grade inflammatory state as a physiological response necessary to restore homeostasis disrupted by environmental factors. Apart from these environmental factors, also genetic factors are of profound importance. Obesity is considered a multifactorial trait, as over 700 genes and chromosomal regions have been specified as having an influence on body weight and metabolic regulation where obesity is only one of the symptoms of congenital genetic disorders or polygenic, associated with the presence of polymorphisms in several genes [2].

The relationship between obesity and an interleukin-mediated inflammatory process has been under investigation for many years. Balistreri et al. [3] believe that the relationship between obesity and obesity-related inflammatory diseases can be explained by evolutionary theory, e.g. the ability to resist hunger and elicit an effective immune response to pathogens. Depending on the severity of metabolic disorders, individuals with obesity can be divided into two categories. The first group includes individuals with mild disorders and low expression of pro-inflammatory factors. On the other hand, in the second group, there is a significant increase in pro-inflammatory factors and we can observe in this group lot of metabolic dysfunction and disorders in processes controlling metabolism. Trayhurn and Wood [4] proposed possible explanations of this situation. The first possibility is the release of pro-inflammatory agents from organs other than white adipose tissue, e.g. the liver. The second theory assumes that white adipose tissue secretes factors stimulating production of inflammatory markers in the liver and other organs. The third possible explanation is that the adipocytes themselves increase production of some or most inflammatory markers. Cellular composition of adipose tissue defines its secretory function. The adipose tissue consists of adipocytes, lymphocytes, macrophages, mast cells, eosinophils, fibroblasts and cells of the blood vessel wall. The number of individual types of cells, their phenotype and distribution determine the type of adipose tissue and the degree of obesity. Macrophages in lean mice and humans make up around 5% of the cells in adipose tissue; during obesity they constitute up to 50% of all adipose tissue cells. Type II macrophages are present in the adipose tissue of lean individuals, while type I macrophages appear in individuals with obesity. Macrophages of type II are responsible for tissue remodelling and inflammation resolution [5]. ‘Classically activated’ M1 macrophages lead to increased expression levels of TNFα and inducible nitric oxide synthase (iNOS) and the release of nitric oxide (NO). The pro-inflammatory cytokines IL12 and IL23 are likewise produced, while synthesis of the anti-inflammatory cytokine IL10 is reduced [6]. Macrophages of type II are involved in the repair of damaged tissues and prevent the development of inflammation. These macrophages have been shown to secrete significant amounts of IL10 with a simultaneous decrease in the synthesis of IL12 and IL23 [7].

The *IL10* gene is located on chromosome 1 at 1q31–1q32, and comprises 4 introns and 5 exons flanked by an untranslated regions (UTR), spanning approximately 5.2 kb [8]. The *IL10* gene is surrounded upstream by other members of the IL10 family of cytokines – *IL19*, *IL20* and *IL24* – and downstream by the *Mapkapk2* gene. *IL10* is expressed in many types of cells within both the innate (including macrophages, monocytes, dendritic cells, mast cells, neutrophils, eosinophils and natural killer cells) and adaptive (including CD4+ T cells, CD8+ T cells and B cells) immune systems [2, 9]. Many studies have shown that polymorphisms in the coding and regulatory non-coding regions of the *IL10* gene have a significant impact on the expression level and functionality of the cytokine IL10, which is associated with susceptibility to the development of a number of diseases based on the inflammatory state. Xia et al. [10] detected a relationship between the rs151811 polymorphism and spastic tetraplegia in patients with cerebral palsy. By contrast, Shahriyari et al. [11] found that the same polymorphism likely plays a protective role in Behcet’s disease. Tsilidis et al. [12] reported evidence for a relationship between the rs3024496 and rs3024498 polymorphisms and colorectal cancer. Lin et al. [13] found a significant association between the rs3024496 genotypes and IBD in children; and an association between the prevalence of the rs3024498 SNP genotype and IBD in adults and children. IL10 may also be involved in control of body mass. Esposito et al. [8] tested premenopausal women with obesity (n = 50) and age-matched women (n = 50) with normal weight. They observed an elevated circulating IL10 level in the obese group; however, low IL10 levels were observed in the group with metabolic syndrome. In another study, Liu et al. [9] observed lower IL10 levels in serum of children with obesity and hypertriglyceridaemia. Immune mechanisms may play a potential role in the development and maintenance of obesity and only a few studies have investigated the relationship between excess body weight and polymorphisms in the *IL10* gene. However, the exact relation between *IL10* gene polymorphisms and the risk of obesity has not been clearly defined.

The aim of this study was to investigate the frequencies of polymorphisms of the *IL10* gene and the relationship between *IL10* gene polymorphisms and the body composition parameters (fat %) and body mass index (BMI) in homogeneous population of physically active overweight and non-overweight young men living in homogeneous environmental conditions.

## MATERIALS AND METHODS

### Study subjects

All participants were recruited from a group of cadets of the Military University. Volunteers and were acquainted with the protocol and research methods. The investigation protocols were performed in accordance with the rules of the World Medical Association Declaration of Helsinki, as well as ethical standards in sport and exercise science research. The procedures were accepted by the Ethics Committee of the Military Institute of Hygiene and Epidemiology – resolution number 07/2018 issued on February 23, 2018. Participants received a written information sheet concerning the study purpose, procedures used, benefits and risks, as well as a consent form.

The study enrolled 131 volunteers — male students aged 19–27 years. Participants filled in the questionnaire screening for exclusion criteria including past diseases, injuries and the presence of severe and chronic pain of any organs or system, both in the past and currently. A general medical examination and electrocardiography (ECG) were performed to confirm the health condition. The cadets participating in the study constituted a homogeneous research group in terms of place of residence (they were stationed on the university campus), nutrition (they were eating at the university canteen) and physical activity (they had similar levels of physical effort exposure due to the training schedule).

Anthropometric measurements and body composition were obtained using standard methods. Height was measured using a portable stadiometer with a precision of 0.1 cm (TANITA HR-001, Tanita Corporation, Japan). Body composition and mass analysis measurements were performed using the TANITA MC-780 analyser (Tanita Corporation, Japan) according to the procedure specified in the instruction manual, as well as characterized by sufficient measuring accuracy and repeatability [14].The assessment of BMI values was made in accordance with the criteria set out by the WHO [1].

The subjects were divided into two groups according to BMI value. The following formula, which is also applied in clinical practice, was used to obtain the BMI value: body mass index (BMI) = body weight/height^2^ (kg/m^2^). The experimental group (OVER_BMI_) enrolled participants with BMI of ≥ 25.0, while the control group (CON_BMI_) consisted of men with BMI values between 20.0 and 25.0 [1]. For further analysis, an additional division was made according to the percentage of fat in total body weight (% fat). The control group (CON_Fat_) consisted of persons with fat content below 20.0%, while the overweight group (OVER_Fat_) was characterized by fat content over 20.0% [15].

All analyses were performed in R version 4.1.2 – SNPassoc, haplo. stats, STAT.package, score test was used to analyse the statistical significance of alleles, genotype was analysed by the likelihood ratio test and Fisher’s test, and haplotypes were analysed by Pearson’s χ^2^ test.

### Genetic analyses

The buccal cell samples were collected with FLOQSwabs swabs (Copan Diagnostics Inc., USA). Genomic DNA was extracted using a High Pure PCR Template Preparation Kit (Roche Diagnostics, Germany). The DNA extraction was performed according to the manufacturer’s instructions. All samples were genotyped using TaqMan assays for *IL10* (rs1518111) C__8828803_1, *IL10* (rs1878672) C__12084302_20, *IL10* (rs3024496) C 15983645_10, *IL10* (rs3024498) C 15983635_10, *IL10* (rs3024505) C 15983681_20 single-nucleotide polymorphisms (SNPs) (Applied Biosystems, USA) on a CFX Connect Real-Time PCR Detection System (Bio-Rad, USA).

### Statistical analysis

Anthropometric data are shown as mean values ± standard deviation. Student’s t-test was used to determine the differences between the experimental groups. Statistical analysis was made using the SNPassoc package for R (version 1.9–2, R Foundation for Statistics Computing, https://cran.r-project.org). Single locus analysis was performed with SNPassoc (version 1.9.2) considering four genetic models (co-dominant, dominant, recessive and overdominant). The models were constructed with respect to the minor allele. The statistical significance of the effect of single alleles on BMI and fat % was calculated using Pearson’s χ^2^ test with the STAT.package. Haplotype analysis was performed with the haplo.stats package. The haplo.score function was used to test the association, with magnitude and direction, between allele combinations with BMI and with fat % under the different models of inheritance (additive, dominant and recessive). Odds ratio was calculated with the haplo.cc statistic and haplotype frequencies with the haplogroup function, and genotype frequencies were analysed using Fisher’s exact test. IBM SPSS Statistics (version 27) was used to calculate differences between groups with the t-test. The level of statistical significance was set at the level of p < 0.05.

## RESULTS

Among 131 investigated individuals, 39 had BMI value exceeding 25 (OVER_BMI_) and for 20 of them fat content exceeded 20% (OVER_Fat_). Mean BMI in OVER_BMI_ was 27.2 ± 1.6 and mean fat share in OVER_Fat_ was 22.9 ± 2.4%. Significant differences (p < 0.05) were found between the control group CON_BMI_ and the study group OVER_BMI_ regarding such features as weight, BMI, fat % and total water content in the body and values of most of these variables established for OVER_BMI_ exceeded the values for CON_BMI_. No significant differences were found for age and height (p = 0.42 and 0.45, respectively). Similarly, significant differences were established for weight, BMI, fat% and total water content between OVER_Fat_ and CON_Fat_. Only values of total water content were lower in OVER_Fat_ than in CON_Fat_, whereas for other variables their values in OVER_Fat_ exceeded values for CON_Fat_ (Table 1).

**TABLE 1:** Anthropometry and body composition of the subjects for BMI and fat %.

Group	All (n = 131)	OVER_BMI_ (n = 39)	CON_BMI_ (n = 92)	OVER_Fat_ (n = 20)	CON_Fat_ (n = 111)
Age	22.4 ± 2.2	22.6 ± 2.5	22.3 ± 2.1	22.30 ± 2.3	22.4 ± 2.2
Height [cm]	179.9 ± 6.6	180.5 ± 7.3	179.6 ± 6.3	181.10 ± 8.3	179.6 ± 6.2
Weight [kg]	78.9 ± 9.8	88.7 ± 8.5*	74.7 ± 6.9*	91.2 ± 9.6*	76.7 ± 8.0*
BMI [pts]	24.3 ± 2.4	27.2 ± 1.6*	23.1 ± 1.4*	27.8 ± 1.9*	23.7 ± 1.8*
Fat %	16.3 ± 4.2	20.9 ± 2.9*	14.3 ± 2.8*	22.9 ± 2.4*	15.1 ± 3.2*
Total water [%]	60.6 ± 3.4	57.1 ± 2.7*	62.1 ± 2.5*	55.5 ± 2.2*	61.5 ± 2.6*

Note:

* statistically significant (p < 0.05) difference in Student T test between CON and OVER groups. BMI – body mass index. CON – control group. OVER – experimental group.

**FIG. 1:**
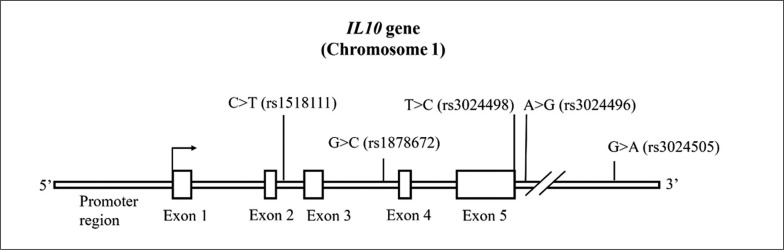
*IL10* gene (Chromosome 1).

Selected *IL10* gene SNPs and their variations are presented in Figure 1 and in Table 2. Genotype frequencies in the OVER_BMI_ group were in Hardy–Weinberg equilibrium, although the observed rs3024498 (*IL10*) genotype frequencies differed significantly from expectations (p = 0.03). The genotype frequency on the border of statistical significance was observed for rs1878672 and rs3024496 for the Hardy-Weinberg equilibrium in the OVER_Fat_ group (p = 0.05 and p = 0.05 respectively) (Table 3).

**TABLE 2:** Selected *IL10* gene single nucleotide polymorphism and their variations.

SNP ID (RefSNPs)	Chromosomal position	Variation	Gene location	Functional consequence
rs1518111	1:206771300 (GRCh38)	c.T > C	Intron	upstream transcript variant
rs1878672	1:206770368 (GRCh38)	c.G > C	Intron	upstream transcript variant
rs3024496	1:206768519 (GRCh38)	c.A > G	non coding transcript	3 prime UTR variant
rs3024498	1:206768184 (GRCh38)	c.T > C	non coding transcript	3 prime UTR variant
rs3024505	1:206766559 (GRCh38)	c.G > A	downstream regulatory sequence	expression influence

Note: RefSNPs – Reference Single Nucleotide Polymorphism.

**TABLE 3:** Minor allele frequencies (MAF) and Hardy-Weinberg expectations (HWE).

SNP	MAF(%)	OVER_BMI_ + CON_BMI_	OVER_BMI_	CON_BMI_	OVER_Fat_	CON_Fat_
***IL10* (rs1518111)**	Allele T 26.72	1	0.66	0.79	0.59	0.81
***IL10* (rs1878672)**	Allele C 45.42	0.16	0.17	0.54	0.05	0.57
***IL10* (rs3024496)**	Allele G 44.66	0.11	0.17	0.30	0.05	0.34
***IL10* (rs3024498)**	Allele C 22.48	0.08	0.03*	0.59	0.54	0.15
***IL10* (rs3024505)**	Allele A 16.79	0.53	0.41	0.18	0.15	0.19

Note:

* statistically significant (p < 0.05). MAF – minor allele frequency.

All *IL10* genotypes (rs1518111, rs1878672, rs3024496, rs3024498 and rs3024505) were tested for potential correlation with BMI. The result of *IL10* rs1518111 is shown in Table 4. The other results are not shown; there was no statistical significance. Analysis of the link between *IL10* rs3024505 and BMI showed a significant association of this SNP with BMI values exceeding 25 (Table 5). Associations were found for the overdominant genetic model (G/G+A/A vs. A/G) with OR values = 0.39, 95% CI 0.16–0.98, p = 0.04 (Table 4). In this model the genotype A/G of rs3024505 was 2.5 times lower than G/G+A/A genotypes (p = 0.06) (Table 5).

**TABLE 4:** Association analysis of the *IL10* gene rs1518111 polymorphism with BMI.

Genotypes and alleles	OVER_BMI_	CON_BMI_
**C/C (%)**	23 (59.0)	47 (51.1)
**C/T (%)**	13 (33.3)	39 (42.4)
**T/T (%)**	3 (7.70)	6 (6.50)
**C (%)**	59 (75.6)	133 (72.3)
**T (%)**	19 (24.4)	51 (27.7)
**OR (95% CI) p-value**		
**Genetic models**		**OVER_BMI_ VS. CON_BMI_**
**Dominant**	C/C vs. C/T + T/T	0.73 (0.34–1.55) p=0.41
**Recessive**	C/C + C/T vs. T/T	1.19 (0.28–5.04) p=0.81
**Overdominant**	C/C + T/T vs. C/T	0.68 (0.31–1.49) p=0.33
**Codominant**	C/C vs. C/T	1.02 (0.23–4.46) p=0.62
	C/C vs. T/T	0.68 (0.31–1.52) p=0.62
**Allelic**	C vs. T	1.19 (0.63–2.33) p=0.68

OR – odds ratio. 95% CI – confidence intervals.

**TABLE 5:** Association analysis between the *IL10* gene rs3024505 polymorphism and BMI.

Genotypes and alleles	OVER_BMI_	CON_BMI_
**G/G (%)**	31 (79.5)	58 (63.0)
**A/G (%)**	7 (17.9)	33 (35.9)
**A/A(%)**	1 (2.60)	1 (1.10)
**G (%)**	69 (88.5)	149 (81.0)
**A (%)**	9 (11.5)	35 (19.0)
**OR (95% CI) p-value**		
**Genetic model**		**OVER_BMI_ vs. CON_BMI_**
**Dominant**	G/G vs. A/G + A/A	0.44 (0.18–1.07) p=0.06
**Recessive**	G/G + A/G vs. A/A	2.39 (0.15–39.28) p=0.55
**Overdominant**	G/G + A/A vs. A/G	0.39 (0.16–0.98) p=0.04
**Codominant**	G/G vs. A/G	0.40 (0.16–1.00) p=0.10
	G/G vs. A/A	1.87 (0.11–30.95) p=0.10
**Allelic**	G vs. A	1.8 (0.7–4.49) p=0.19

OR – odds ratio. 95% CI – confidence intervals.

The same analysis was performed for the association between *IL10* gene polymorphisms and fat %. The only significant association was found for *IL10* (rs3024505) in the overdominant model (G/G+A/A vs. A/G) with OR = 0.21, 95% CI 0.05–0.97, p = 0.02 (Table 6).

**TABLE 6:** Association analysis of the *IL10* gene rs3024505 polymorphism with fat %.

Genotypes and alleles	OVER_Fat_	CON_Fat_
**G/G (%)**	17 (85.0)	72 (64.9)
**A/G (%)**	2 (10.0)	38 (34.2)
**A/A(%)**	1 (5.00)	1 (0.90)
**G (%)**	36 (90.0)	182 (82.0)
**A (%)**	4 (10.0)	40 (18.0)
**OR (95% CI) p-value**		
**Genetic model**		**OVER_Fat_ vs. CON_Fat_**
**Dominant**	G/G vs. A/G + A/A	0.33 (0.09–1.18) p=0.06
**Recessive**	G/G + A/G vs. A/A	5.79 (0.35–96.57) p=0.25
**Overdominant**	G/G + A/A vs. A/G	0.21 (0.05–0.97) p=0.02
**Codominant**	G/G vs. A/G	0.22 (0.05–1.02) p=0.05
	G/G vs. A/A	4.24 (0.25–71.18)p=0.32
**Allelic**	G vs. A	1.97 (0.65–8.06) 0.31

OR – odds ratio. 95% CI – confidence intervals.

In the codominant model the genotype A/G of rs3024505 was 4.5 times lower than the G/G genotype (p = 0.06) in the OVER_Fat_ group. In the overdominant model the genotype A/G was over 4.7 times lower than G/G+A/A (p = 0.03) (Table 6).

Polymorphisms *IL10* rs1518111, *IL10* rs1878672, *IL10* rs3024496, *IL10* rs3024498 and *IL10* rs3024505 haplotypes of these polymorphisms were analysed. Only haplotypes with frequency over 5% were considered. Most common haplotype was CGATG (0.26%, *IL10* (rs1518111) C > T, *IL10* (rs1878672) G > C, *IL10* (rs3024496) A > G, *IL10* (rs3024498) T > C and *IL10* (rs3024505) G > A). A statistically significant association with BMI was found for CCGTA haplotype in the additive (score = -2.00, p = 0.04) and dominant model (score = -2.30, p = 0.02). No significant association was found for haplotypes and fat %. For CCGTA haplotype the chance of being CON_BMI_ was over 2 times greater than being OVER_BMI_ (Table 7).

**TABLE 7:** Haplotypes distribution and its association of *IL10* with BMI.

Haplotypes						Haplotype Frequencies %					additive(score = 17.00, p-value = 0.01)		dominant (score = 14.09, p-value = 0.03)		recessive (score = 0.43, p-value = 0.93)	
*IL10* rs1518111	*IL10* rs1878672	*IL10* rs3024496	*IL10* s3024498	*IL10* rs3024505	OVER_BMI_ + CON_BMI_	OVER_BMI_	CON_BMI_	OR	95% CI		score	p-value	score	p-value	score	p-value
C	C	G	T	A	0.16	0.09	0.18	0.44	0.16	1.21	-2.00	**0.04**	-2.30	**0.02**	NA	NA
T	G	A	T	G	0.24	0.22	0.26	0.72	0.32	1.60	-0.66	0.51	-1.00	0.33	0.24	0.81
C	C	G	C	G	0.23	0.21	0.23	0.74	0.35	1.58	-0.33	0.75	-0.60	0.59	0.24	0.81
C	C	G	T	G	0.06	0.07	0.06	1.10	0.36	3.35	0.38	0.70	-0.40	0.66	NA	NA
T	G	A	C	G	0.02	0.02	0.02	1.49	0.17	13.41	0.65	0.52	0.65	0.52	NA	NA
C	G	A	T	G	0.26	0.27	0.24	1.00	NA	NA	0.65	0.51	0.57	0.57	0.50	0.62

OR – odds ratio. 95% CI – confidence intervals. NA – not applicable.

## DISCUSSION

The most important findings of this study are association between BMI and CCGTA haplotype in the additive model (score = -2.00, p = 0.04) and in the dominant model (score = -2.30, p = 0.02). The obtained results indicate a statistically significant contribution of the selected *IL10* haplotype of polymorphisms in the regulation of body weight in physically active individuals. For the first time polymorphisms of the *IL10* gene (rs1518111, rs1878672, rs3024496, rs3024498 and rs3024505) have been studied in the context of the BMI and body composition parameters (fat %) in physically active young men. A significant association was found between selected *IL10* SNP polymorphisms haplotypes and increased BMI and body fat percentage. The excess body weight may increase the risk of the development of chronic inflammation accompanied by the secretion of many pro-inflammatory factors. It has been observed that adipose tissue in obese individuals secretes mainly proinflammatory cytokines, i.e. TNF, IL6, leptin, visfatin, resistin, angiotensin II and plasminogen activator inhibitor 1, whereas adipose tissue of lean individuals secretes anti-inflammatory adipokines such as adiponectin, as well as transforming growth factor (TGF), interleukin (IL) 10, IL4, IL13, IL1 receptor antagonist (IL1RA) and apelin [16, 17]. Polymorphisms in the genes of pro- and anti-inflammatory cytokines and/or their receptors may exacerbate cytokine imbalances and thus contribute to the development or worsening of obesity [18]. A wide range of pro- and anti-inflammatory cytokines have been analysed in relation to obesity [16, 18, 19, 20], including some information regarding IL10 [8, 11, 17].

Single nucleotide polymorphisms (SNPs) in cytokine genes or regions in their close proximity are considered to be important in genetic control of the production of cytokines, including IL 10. IL10 as anti-inflammatory cytokine plays an important role in the regulation of the immune system by decreasing cytokine production, inhibiting matrix-degrading metalloproteinases, and promoting the switching of lymphocytes to the Th2 phenotype [21]. Furthermore, IL10 exerts essential control over the biochemical parameters such as LDL, VLDL, HDL, triglycerides and glucose level [22]. An increase in *IL10* expression was observed in adipose tissue of obese humans and rodents, which is consistent with the finding that obesity is associated with increased levels of circulating IL10 [8]. Hence, it is important to identify and characterize the regulation of IL10 in obesity, especially as it has been shown that polymorphism of genes encoding interleukins may contribute to an increased risk of obesity [21].

Our research showed the possible relationship of the haplotype CCGTA with maintaining normal body weight, as its incidence in the CON_BMI_ group was more than two times higher than in the OVER_BMI_ group (additive model p = 0.04 and dominant model p = 0.02). These data support previous reports that polymorphisms and allele variants of cytokine genes may be associated with obesity and may play an important role in body weight regulation.

Our association analysis of the *IL10* gene rs3024505 polymorphism with body fat percentage was statistically significant in the overdominant model (p = 0.02). In-depth tests showed that the odds of being OVER_Fat_ for A/G were over 4.7 times lower than for G/G-A/A (p = 0.02) in the overdominant model.

One of the analysed polymorphisms, 1170 C/T rs1518111, has been previously linked to reduced expression of *IL-10* mRNA, has an allelic association with several other variants of *IL10* polymorphisms and is an indicator of poor outcome and enhanced systemic inflammation in patients with acute coronary syndrome [23]. The polymorphism *IL10* rs3024505 has been associated with a predisposition to inflammatory bowel disease development, e.g. ulcerative colitis, Crohn’s disease; and sensitivity to Sjögren’s syndrome [13, 24–30]. Similar developments were observed during analysis of the *IL10* SNP rs3024496, where it has been shown to be associated with the inflammatory response or decreased production of IL10 by peripheral blood leukocytes, and with prostate and colorectal cancer [12, 31, 32]. Based on the data above, we set out to extend and validate the theory in which inflammation-related *IL10* polymorphisms affect body weight parameters, with an emphasis on obesity, in physically active young men. Tan et al. [33] summarized the information in 36 articles, with a total of 15 913 overweight participants/participants with obesity, and described the possible association of various genes with weight control. An association has been demonstrated between genes involved in energy homeostasis, adaptive thermogenesis, lipoprotein metabolism, appetite regulation and the insulin signalling pathway, and obesity, and most interventional studies have focused on the effect of single candidate gene variants on weight loss. Further large-scale research is needed to evaluate the impact of many candidate genes. A healthy lifestyle, a balanced diet, and regular physical activity will benefit persons who carry the risk allele of obesity-related genes [34].

Genetic polymorphisms related to the yo-yo effect are controversial due to design differences between studies [33]. Genetic risk can be used to determine appropriate weight loss strategies and longterm weight maintenance. This can result in a reduction in the incidence of obesity, a reduction in the risk of obesity-related diseases and mortality, and a reduction in the economic burden of obesity.

As both environmental and genetic factors are involved in the pathogenesis of obesity, environmental factors may mask genetic factors, which may be critical to the results of genetic analysis. In the conducted research, the influence of the environment was reduced due to the controlled and standardized conditions in which the individuals under analysis were living.

The experiments in this study were conducted with a relatively small sample size, which can be considered as a limitation, but the high level of homogeneity increases the relevance of this study. This allowed us to assess the association of the studied *IL10* gene SNPs on body weight gain while excluding many environmental factors that could limit the value of the obtained genetic results. The participants were relatively young, healthy, and physically active, which may have influenced the results because systematic exercise reduces body weight and body fat. Also, the analysed subjects were fed according to the army’s nutrition standards, taking in consideration their age and physical exertion. Despite the small sample size, which is the main limitation, a statistically significant trend was observed which may indicate a crucial effect of haplotype CCGTA [34,35].

## LIMITATIONS

In the pathogenesis of obesity both genetic and environmental factors play a significant role [36]. Due to the extensive influence of various environmental factors, determining the influence of genetic factors on obesity or overweight susceptibility can be complicated. To limit the influence of varying environmental factors, this study selected a fairly homogeneous and environmentally isolated group of individuals. The study group consisted of unrelated male military cadets based in dormitories of a military university in Warsaw. All of them have undergone fitness tests both during recruitment to the military college and each year during their service, to ensure an appropriate level of physical fitness. Moreover, sports activities were an essential part of their education and daily routine. They usually ate their meals in the university canteen, so their habitual diet was not diverse. Provided meals were balanced in terms of nutritional value and energetic value and they complied with the nutritional recommendation for military professionals. In the study group only 29.8% of participants had values of BMI exceeding 25. Excessive storage of fat in the body exceeding 20% was observed only in 15.3% of participants. Although the size of the study group was rather small, its homogeneity allowed us to reduce the influence of environmental factors, which increased the significance of the analysis.

## CONCLUSIONS

The present results indicate that the selected *IL10* polymorphisms are involved in the regulation of body weight in physically active individuals. Analysis of the association between *IL10* rs3024505 genotype and BMI showed a significant correlation of this SNP with a BMI value over 25, whereas rs1518111, rs1878672, rs3024496, rs3024498 *IL10* genotypes showed no significant association with these indicators. A statistically significant association with BMI was found for CCGTA haplotype (rs1518111 C > T, rs1878672 G > C, rs3024496 A > G, rs3024498 T > C and rs3024505 G > A respectively). The obtained results extend and lend statistical credence to the theory that inflammation-related cytokine *IL10* polymorphisms influence body weight parameters, with particular emphasis on obesity, in physically active young men.

## Institutional Review Board Statement

Procedures used for this study were conducted in accordance with the World Medical Association’s Declaration of Helsinki, and the protocol was approved by the Ethics Committee of the Military Institute of Hygiene and Epidemiology—resolution number 07/2018, dated 23.02.2018.

## Informed Consent Statement

Informed consent was obtained from all subjects involved in the study.

## Data Availability Statement

The data presented in this study are available on request from the corresponding author. The data are not publicly available due to ethical reasons.

## Funding

The study was financed by the Ministry of Science and Higher Education in 2020/2022 as part of the Scientific School of the Academy of Physical Education in Warsaw—SN No. 5 “Biomedical determinants of physical fitness and sports training in adult population” and by grant “KOSCIUSZKO” II edition, decision no. 509/2017/DA from the Ministry of National Defence.

## Conflicts of Interest

The authors declare no conflict of interest.
